# The Influence of Surface Wettability and Topography on the Bioactivity of TiO_2_/Epoxy Coatings on AISI 316L Stainless Steel

**DOI:** 10.3390/ma12111877

**Published:** 2019-06-11

**Authors:** Aleksandra Kocijan, Marjetka Conradi, Matej Hočevar

**Affiliations:** Institute of Metals and Technology, Lepi pot 11, SI-1000 Ljubljana, Slovenia; marjetka.conradi@imt.si (M.C.); matej.hocevar@imt.si (M.H.)

**Keywords:** epoxy coatings, TiO_2_ nanoparticles, biocompatibility, antibacterial properties

## Abstract

Epoxy/TiO_2_/epoxy and epoxy/FAS-TiO_2_/epoxy coatings were applied to the surface of AISI 316L stainless steel with the aim to improve the biocompatibility and antibacterial properties. Contact-angle measurements were used to evaluate the wetting properties of the epoxy, epoxy/TiO_2_/epoxy and epoxy/FAS-TiO_2_/epoxy coatings. The epoxy and epoxy/TiO_2_/epoxy coatings were hydrophilic compared with the strongly hydrophobic epoxy/FAS-TiO_2_/epoxy coating. The average surface roughness (*S_a_*) of the epoxy/FAS-TiO_2_/epoxy coating was higher than that of the epoxy/TiO_2_/epoxy coating due to the formation of agglomerates. The biocompatibility evaluation revealed that the cell attachment was significantly higher on the epoxy/FAS-TiO_2_/epoxy and epoxy/TiO_2_/epoxy coatings compared with the pure epoxy coating. We also observed improved antibacterial properties for the epoxy coatings with the addition of both TiO_2_ and FAS-TiO_2_ nanoparticles.

## 1. Introduction

Improved cell attachment and resistance to bacterial adhesion are two critical characteristics to consider in an effective biomaterial design. The appropriate surface modification of the substrate is therefore crucial for a desirable biological response. Various techniques, such as the application of polymer coatings [1], electron-beam lithography [2], plasma treatment [3,4], self-assembly techniques [5] and laser surface processing [6], were introduced to tune the surface properties in order to enhance the cell attachment and/or to prevent the bacterial adhesion. When a biomaterial is exposed to physiological conditions, multiple aspects of the surface properties, including wettability, surface energy and topography, need to be evaluated.

The biocompatibility of a material is related to cell behaviour on contact with the surface where the surface characteristics of materials such as surface topography, chemistry or surface energy, play an essential part in the adhesion process. The quality of this first phase of the cell-material interaction influences and enables good proliferation and differentiation of the cells on the surface [7]. This morphological transformation of the cells with time can be explained through a re-arrangement of the focal contacts and the intracellular cytoskeleton [8]; therefore, it is very important to optimize the surface properties to achieve good adhesion. Material biocompatibility is significantly influenced by the surface topography, as cell attachment and proliferation can be controlled by micro/nanoscale roughness through mimicking the natural environment at the molecular level [9]. Different topographic patterns influence the size, shape and spatial distribution of the attached cells [10]. In addition, the wettability also plays an important role in cell adhesion [11]. It has been shown that surfaces with moderate wettability have a higher rate of cell attachment than superhydrophilic and superhydrophobic surfaces [12,13].

Furthermore, the effect of surface roughness as a critical factor that directly influences bacterial adhesion has also been widely studied [14]. It has been shown that interactions between topographic patterns and bacterial adhesion cannot be generalised, as the size and the shape of the bacteria are crucial for their spatial distribution. An increased surface roughness has been shown to associate with improved cell integration. Additionally, most studies indicate a positive correlation between a large surface area with a rough topography and the amount of adhering bacteria [13,15]. Antibacterial applications are often based on TiO_2_ coatings, which are based on the photocatalytic activity of anatase TiO_2_ [16,17,18]. Due to its photocatalytic properties, TiO_2_ is used in many applications where surfaces cannot be cleaned with conventional methods and have to inhibit the growth of pathogenic bacteria [16]. Due to the high stability of TiO_2_ with steady antibacterial activity, even under long-term exposure to UV, such coatings are highly applicable for self-cleaning applications [17]. In addition, among several antimicrobial nanocrystalline materials, TiO_2_ is the most benign with respect to the environment and human health [18].

In our study, the surface topography and wettability were controlled by preparing TiO_2_/epoxy coatings with as-received and fluoroalkylsilane (FAS) functionalised TiO_2_ nanoparticles. We used TiO_2_ nanoparticles in the form of anatase, which are suitable for applications in self-cleaning and antibacterial coatings due to their photocatalytic properties [19]. We studied the effect of the surface properties on the biocompatibility and antibacterial properties of TiO_2_/epoxy coatings on AISI 316L stainless steel. We correlated the surface properties with the amount of attached cells and/or adhering bacteria. The biocompatibility of the coatings was evaluated by bone osteosarcoma cell attachment and the antibacterial properties by the adhesion of *Escherichia coli* (*E. coli*).

## 2. Materials and Methods

Substrate.—Austenitic stainless steel AISI 316L (17% Cr, 10% Ni, 2.1% Mo, 1.4% Mn, 0.38% Si, 0.041% P, 0.021% C, <0.005% S in mass fraction) was used as a substrate. Discs of 25-mm diameter were grinded with SiC emery paper up to 4000 grit, diamond polished up to 1 µm, ultrasonically cleaned with ethanol and dried in warm air.

Coating preparation.—Two sets of coatings were prepared on the AISI 316L substrate with as-received TiO_2_ nanoparticles and fluoroalkylsilane (C_16_H_19_F_17_O_3_Si, FAS) functionalised TiO_2_ nanoparticles in the form of a sandwich structure: Epoxy/TiO_2_/epoxy coating and epoxy/FAS-TiO_2_/epoxy coating. In the first step, we applied biocompatible epoxy (USP Class VI, two-component EPO-TEK 302-3M, EPOXY TECHNOLOGY, Inc., Billerica, MA, USA), which was mixed in the wt% ratio 100:45, spin-coated onto the substrate and cured for 3 h at 65 °C. In the next step, two sizes of TiO_2_ nanoparticles, 30-nm (Cinkarna Celje) and 100-nm (US Research Nanomaterials, Inc.) were applied to achieve a dual-size effect. Three separate deposits of 20 μL of 3 wt% TiO_2_ nanoparticle ethanol solution (30 nm and 100 nm) were alternately spin-coated on a primary epoxy layer. The coating was finalised with the top epoxy layer. The same procedure was repeated with the functionalized TiO_2_ nanoparticles, which were prepared in 1 vol% fluoroalkylsilane (C_16_H_19_F_17_O_3_Si, FAS) ethanol solution. The reference coating was pure epoxy coated on the AISI 316L substrate.

Surface roughness.—An optical 3D metrology system, Alicona Infinite Focus (Alicona Imaging GmbH), was employed for the surface-roughness analysis. Three measurements per sample were performed at a magnification of 20× with a lateral resolution of 0.9 μm and a vertical resolution of about 50 nm. The IF-MeasureSuite (Version 5.1) software was used to evaluate the average surface roughness, *S_a_*:

(1) $$Sa=\frac{1}{L_{x}}\frac{1}{L_{y}}\int_{0}^{L_{x}}\int_{0}^{Ly}\left|z(x,y)\right|dxdy$$

where *L_x_* and *L_y_* are the acquisition lengths of the surface in the *x* and *y* directions and *z*(*x*,*y*) is the height. The size of the analysed area was 714 × 542 μm^2^.

Contact-angle measurements.—The static water contact-angle measurements on the epoxy-, epoxy/TiO_2_/epoxy- and epoxy/FAS-TiO_2_/epoxy-coated AISI 316L substrate were performed using a surface-energy evaluation system (Advex Instruments s.r.o., Brno, Czech Republic). The volume of the water droplets of 5 μL was applied and the average contact angle was determined from five measurements on each surface using a Young-Laplace fitting. The measurements were carried out at 20 °C and ambient humidity.

Cell adhesion and viability.—Bone osteosarcoma cells (MG-63; (ATCC^®^ CRL-1427™, Krasteva Institute of Biophysics and Biomedical Engineering, Bulgarian Academy of Sciences, Sofia, Bulgaria)) were used for the assessment of the cytocompatibility/biocompatibility on sterilized epoxy-, epoxy/TiO_2_/epoxy- and epoxy/FAS-TiO_2_/epoxy-coated steel substrates. The MG63 cells were cultured under controlled conditions (37 °C, 5% CO_2_, high humidity) in Dulbecco’s modified Eagle’s medium (DMEM), supplemented with fetal bovine serum (FBS; 10%, v/v) and 4-mM L-glutamine (all the reagents were purchased from Sigma-Aldrich, Darmstadt, Germany). The cells were sub-cultured once a week or when they reached 65–70% confluence. Before harvesting with Trypsin-0.25% EDTA (Sigma-Aldrich, Germany) for approximately 10 min at 37 °C, the cells were washed three times with Phosphate Buffered Saline without Ca^2+^ and Mg^2+^ (PBS; Sigma-Aldrich, Germany). The cells were then resuspended in the growth medium, centrifuged at 200 g for 5 min, and plated at a seeding density of 3 × 10^4^ cells/cm^2^ in 6-well plates (Sigma-Aldrich, TPP*®*, Germany) containing the studied specimens, all in 3 mL of growth medium (27 × 10^4^ cells/well) and incubated for 24 h under controlled laboratory conditions (5% CO_2_/95% air at 37 °C).

After a 24-h incubation, the samples with cells were rinsed with Dulbecco’s Phosphate-Buffered Saline and stained with 2-μg/mL Hoechst 33342 (stains the nuclei of all the cells blue) and 2-μg/mL Propidium iodide (stains the nuclei of the non-viable cells red) for 20 min. Fluorescent microscope (Axio Imager.Z1; Carl Zeiss, Jena, Germany) was used to observe and count stained cells. Twenty images per sample were randomly taken at 100× magnification. The free software program ImageJ was used for quantitative image analyses of the density of the attached cells and the cell viability. The results were normalized to the sample’s surface area.

Scanning Electron Microscopy (SEM).—SEM analysis using a FE-SEM JEOL JSM-6500F (Tokyo, Japan) was employed to investigate the surface morphology of the coatings as well as the distribution, shape and morphology of the attached M63 cells. Prior to SEM imaging, the specimens were processed for fixation. After 24 h of incubation, the cell-culture medium was removed, the cells were washed three times with PBS and fixed for 24 h at 4 °C using a modified Karnovsky fixative, composed of 2.5% glutaraldehyde (SPI Supplies, West Chester, PA, USA) and 0.4% paraformaldehyde (Merck, Darmstadt, Germany) in a 0.1-M Na-phosphate buffer (NaH_2_PO_4_ · 2H_2_O and Na_2_HPO_4_·2H_2_O; all the chemicals from Merck, Germany). The samples were washed in the buffer for 3 × 10 min and post-fixated with 1% osmium tetroxide (OsO_4_) (SPI Supplies, USA; 1 × 60 min), followed by washing in the buffer for 3 × 10 min. The samples were dehydrated through an alcohol gradient (30%–100%) (EtOH; Merck, Germany) with each step lasting 10 min. Further dehydration steps were conducted with a mixture of Hexamethyldisiloxane (HMDS; SPI Supplies, West Chester, PA, USA) and absolute EtOH (1:1; v/v; 10 min), 3:1 (HMDS: absolute EtOH, v/v; 10 min) and absolute HMDS (10 min) and HMDS (2 h), which was finally left to evaporate.

Statistical evaluation.—The experiments were made in triplicate and 20 images per sample (n = 60 per surface coating) were randomly taken and the results from the cell adhesion, the viability assay and the biocompatibility were expressed as the arithmetic mean ± standard deviation (SD). The data were tested with the non-parametric Mann-Whitney U test and the calculations were performed with Statgraphics Plus 4.0. All the data were submitted for an analysis of variance (ANOVA) and Duncan’s multiple range test, where appropriate.

Antibacterial evaluation.—The antibacterial activity of the treated material was determined as a decrease in the proportion of attached cells. *E. coli* (DSM 1576) was grown overnight in TSB (tripton soy bujon), diluted to 0.5 McFarland and 1 mL applied on each tested plate. After 4 h of incubation at 37 °C the plates were washed twice with a sterile physiological solution. To remove the bound bacterial cells the plates were exposed to sonication (60 s); (IKA, Yellowline). The obtained sonicate was tenfold diluted and appropriate dilutions inoculated on TSA in parallel. After 24 h of incubation at 37 °C the colony-forming units were counted.

## 3. Results and Discussion 

### 3.1. Surface Properties

**Figure 1:**
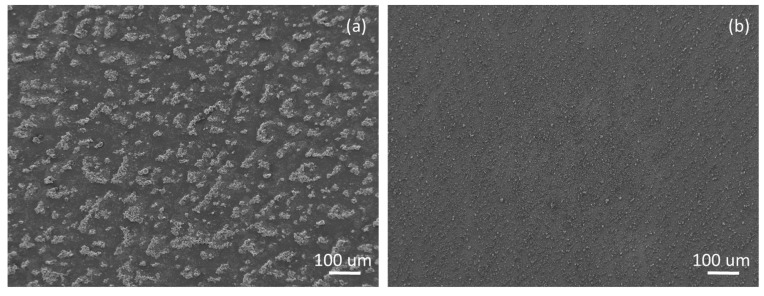
SEM images of the surface morphology of (**a**) epoxy/FAS-TiO_2_/epoxy and (**b**) epoxy/TiO_2_/epoxy coatings on AISI 316L stainless steel.

Figure 1 reveals the morphology of the epoxy/TiO_2_/epoxy and epoxy/FAS-TiO_2_/epoxy coatings. The main difference between the two coatings was in the length scales of the average size of the nanoparticle agglomerates. An epoxy/FAS-TiO_2_/epoxy coating is characterised by the formation of agglomerates up to a few tens of microns (Figure 1a). In contrast, in an epoxy/TiO_2_/epoxy coating, the nanoparticles are more finely dispersed and smaller agglomerates are observed, typically of a few microns (Figure 1b). The morphological differences between the two coatings can be attributed to the compatibility of the TiO_2_ nanoparticles with epoxy resin. The as-received TiO_2_ nanoparticles are hydrophilic and are therefore compatible with the hydrophilic nature of epoxy resin, which enables a more homogeneous distribution of the nanoparticles in the polymer matrix. FAS-functionalisation of the TiO_2_ nanoparticles results in the superhydrophobic nature of the nanoparticles, which causes agglomeration and prevents an even distribution in the hydrophilic polymer matrix [20].

The surface wettability was analysed using five static water contact-angle measurements on different spots all over the sample. The results showed (Table 1) that the epoxy coating and the epoxy/TiO_2_/epoxy coating were hydrophilic, with contact angles of 75° and 83°, respectively. The epoxy/FAS-TiO_2_/epoxy coating was, on the other hand, strongly hydrophobic, with a contact angle of 120°.

The surface energies were calculated using an equation-of-state approach [21,22]:

(2) $$\cos\theta =\;-1+2\sqrt{\frac{\gamma_{s}}{\gamma_{l}}\;}e^{-\beta {\left(\gamma_{s}-\gamma_{l}\right)}^{2}}$$

For a given value of the surface tension of a probe liquid *γ_l_* (i.e., for water *γ_l_* = 72.8 mN/m) [23] and *θ*^W^ measured on the same solid surface, the constant β and the solid surface tension *γ_s_* values were determined using the least-squares technique. For the fitting with Equation (2), a literature value of β = 0.0001234 (mJ/m^2^)^−2^ was used, as weighted for a variety of solid surfaces. The calculated values for the solid surface energy are listed in Table 1. We can see that the surface energy decreased with the increased hydrophobicity of the coatings, which is important for the evaluation of the biocompatibility and antibacterial properties of the coatings.

The average surface-roughness parameter *S_a_* was used to evaluate the morphology difference between the three coatings under investigation. As shown in Table 1, the epoxy-coated steel substrate is smooth with an average surface roughness of 50 nm. The rougher epoxy/FAS-TiO_2_/epoxy surface is covered with large agglomerates, exhibiting high *S_a_* = 600 nm. The epoxy/TiO_2_/epoxy coating, on the other hand, results in a reduced average surface roughness of 320 nm compared with the epoxy/FAS-TiO_2_/epoxy surface due to more finely dispersed nanoparticles with no visible agglomerates.

### 3.2. Biocompatibility Evaluation

**Figure 2:**
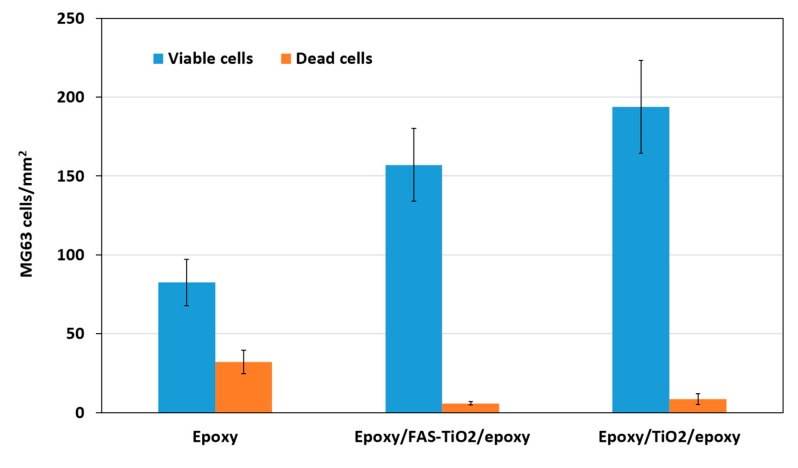
Adhesion of MG63 cells to epoxy, epoxy/FAS-TiO_2_/epoxy and epoxy/TiO_2_/epoxy coatings after 24 h of incubation, mean values (+SD) of attached cells/mm^2^. Number of attached viable cells on epoxy/FAS-TiO_2_/epoxy and epoxy/TiO_2_/epoxy was higher and the number of dead cells was significantly lower compared with the pure epoxy coating.

**Figure 3:**
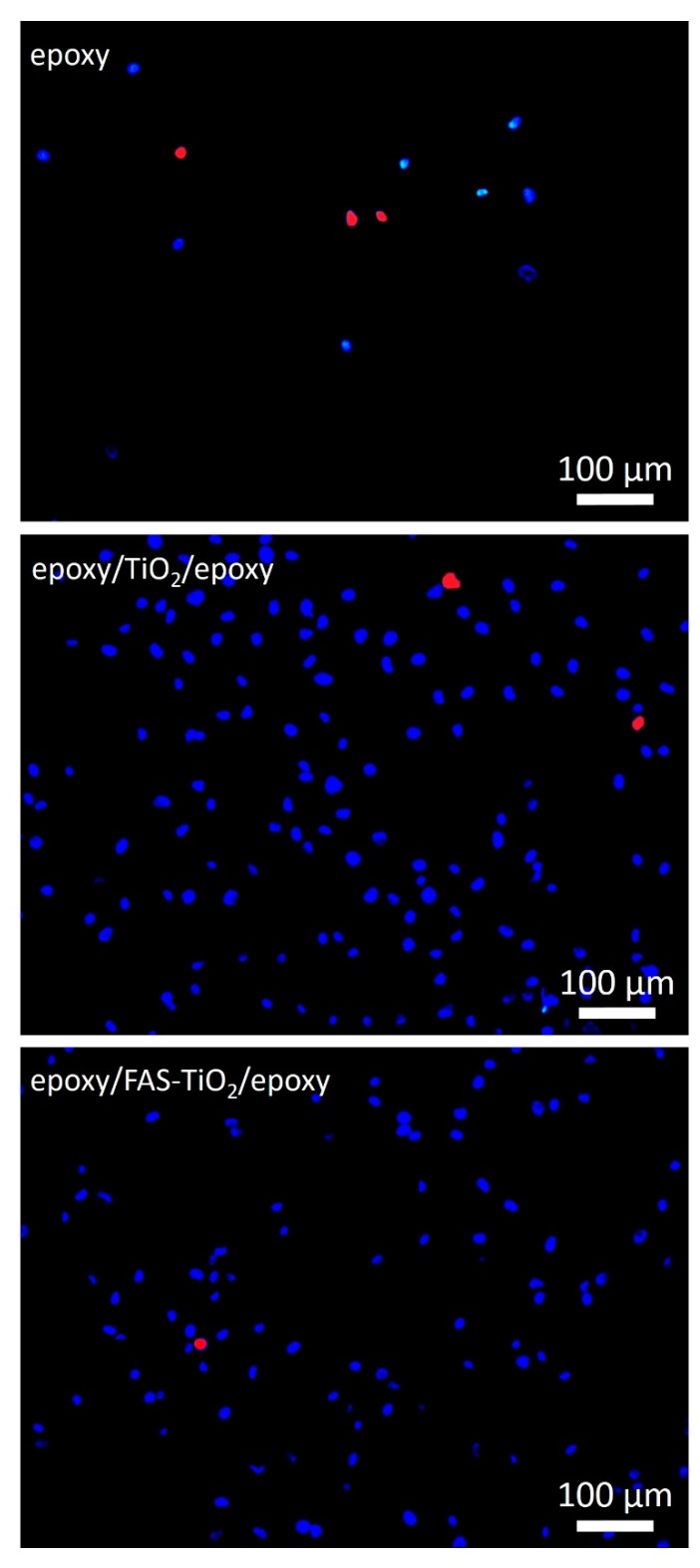
Representative fluorescence-microscopy images showing the number of viable (blue—nuclei of viable cells) and dead cells (red—nuclei of dead cells) on epoxy, epoxy/FAS-TiO_2_/epoxy and epoxy/TiO_2_/epoxy samples after 24 h of incubation.

The cell adhesion, viability and cell morphology of the MG63 cells on epoxy, epoxy/FAS-TiO_2_/epoxy and epoxy/TiO_2_/epoxy samples were investigated. The surface characteristics of the materials play an important part in the adhesion process and, consequently, the adhesion phase of the cell–material interaction influences the proliferation and differentiation of the cells on the surface [7]. We observed that the number of attached viable osteoblast cells varied on different substrates and the attachment was significantly higher on the epoxy/FAS-TiO_2_/epoxy- and epoxy/TiO_2_/epoxy-coated, compared with pure epoxy-coated, samples after 24 h of exposure. The epoxy/TiO_2_/epoxy coating showed a higher degree of cell adhesion compared with the epoxy/FAS-TiO_2_/epoxy coating (Figure 2). However, higher adhesion on the surface does not necessarily suggest that the cells are viable or functional. Therefore, fluorescence microscopy was used to observe the viability of the adhered cells. According to the fluorescence images, the number of dead cells observed on both coatings with TiO_2_ nanoparticles was significantly lower (5.9 ± 0.5 cells/mm^2^ on the epoxy/FAS-TiO_2_/epoxy and 8.7 ± 3.5 cells/mm^2^ on the epoxy/TiO_2_/epoxy) compared with the pure epoxy coating (32.1 ± 7.3 cells/mm^2^) (Figure 2 and Figure 3).

SEM was used to assess the attachment and morphology of the MG63 cells on all the investigated samples. There were no differences observed in the attachment pattern between the different samples. The cells were randomly attached without any particular direction on the epoxy as well as on the rougher epoxy/FAS-TiO_2_/epoxy and epoxy/TiO_2_/epoxy coatings.

The cell morphology was analysed in order to understand the cells’ response to the coating. Flattened and well-spread polygonal cells with extended filopodia represent the normal morphology of healthy, well-adhered cells with a high cellular interaction with the substrate compared to the smaller, round-shaped morphology of the non-proliferating/apoptotic-like cells, indicating poor adhesion [24,25].

**Figure 4:**
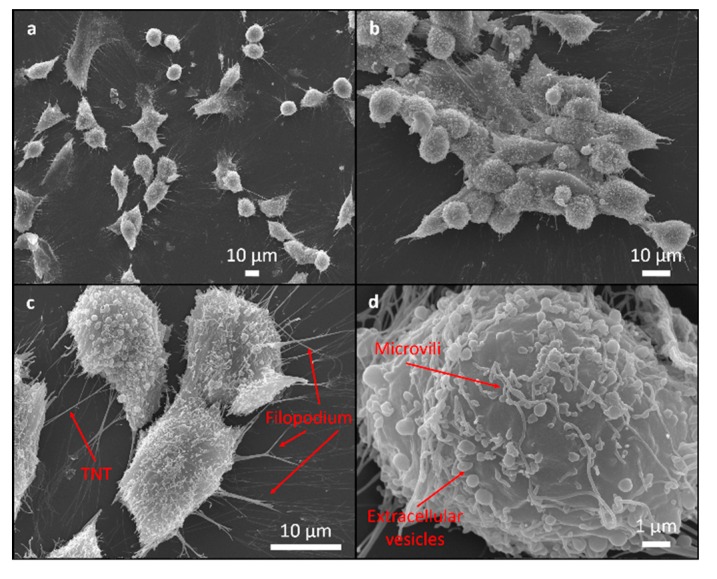
SEM images of MG63 cells adhering to the epoxy surface. Cell adhesion pattern (**a**), round cell adhesion on the top of the other flat cells and in agglomerated form (**b**), surface morphology with filopodia attachment to the surface and cell connection tunneling nanotube (TNT) for intercellular communications between adjacent cells (**c**) and surface morphology with extracellular vesicles and microvilli (**d**).

**Figure 5:**
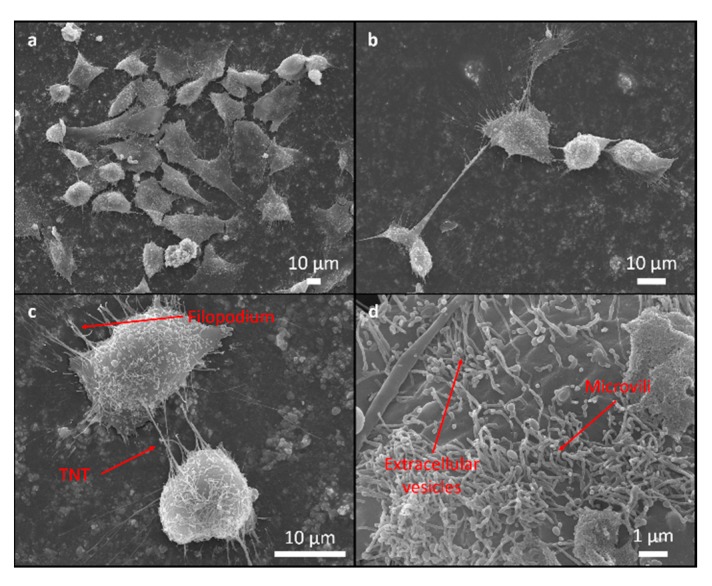
SEM images of MG63 cells adhering to epoxy/FAS-TiO_2_/epoxy surface. Cell adhesion pattern (**a**), intercellular communications not only between adjacent cells but also among cells far from each other (**b**), filopodium anchoring to the surface and intercellular communications between adjacent cells (**c**) and surface morphology with extracellular vesicles and microvilli (**d**).

**Figure 6:**
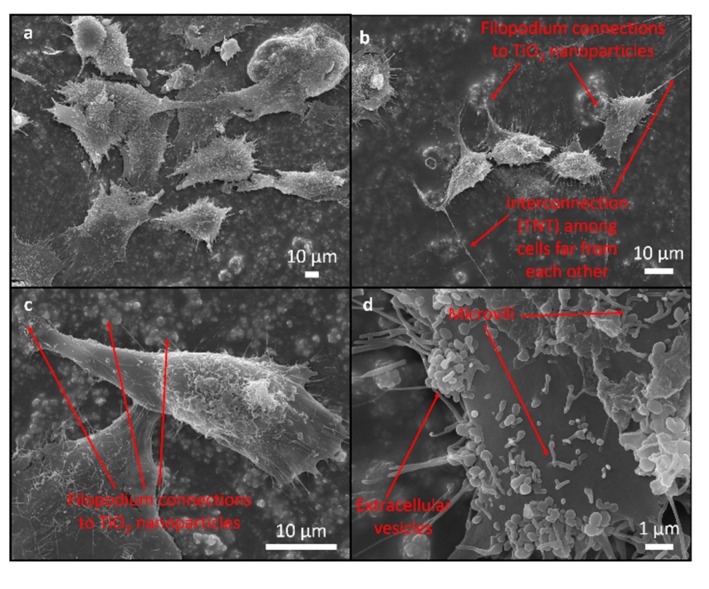
SEM images of MG63 cells adhering to the epoxy/TiO_2_/epoxy surface. Cell adhesion pattern (**a**), filopodia connections to TiO_2_ nanoparticles and intercellular communications between cells far from each other (**b**), filopodium anchoring to the TiO_2_ nanoparticles on the surface (**c**) and high-magnification image of extracellular vesicles and microvilli on the membrane surface (**d**).

The majority of the cells cultured for 24 h were polygonal and they differed in terms of size and the degree of flatness, with the filopodia attachment to the surface suggesting a high cellular interaction with the coating and the indication of normal growth (Figure 4, Figure 5 and Figure 6). The percentage of rounded, non-flattened cells, indicating poor adhesion and low surface interactions, was the lowest on the epoxy/FAS-TiO_2_/epoxy (5 ± 2%) and the highest on the epoxy coatings (10 ± 4%). The size of the polygonal cells was mainly 30 ± 8 μm. The round-shaped cells were smaller (10 ± 3 μm) and agglomerated on top of the other flat cells. Mostly, they were not firmly attached to the surface, which indicates that they did not find a proper site or contact to start the adhesion process and proliferation. On rougher epoxy/FAS-TiO_2_/epoxy and epoxy/TiO_2_/epoxy coatings, the filamentous protrusions (filopodia) found TiO_2_ nanoparticles as the anchoring points for the adhesion (Figure 5 and Figure 6).

SEM images also reveal that the cell’s outer membrane is covered with microvilli, extracellular vesicles (EVs) and cell interconnections known as tunneling nanotubes (TNTs), not only between the adjacent cells but also among the cells far from each other (Figure 4, Figure 5 and Figure 6).

A heterogeneous collection of EVs, exosomes and ectosomes, generated by all the cells, differ in size, composition, mechanisms of assembly, regulation of release and play an important role in intercellular communications [26,27,28]. Like the EVs, also the TNTs, long thin membranous structures that connect two or more cells, enable intensive intercellular communications through a direct membrane cell-cell interaction as well as the transfer of various material and cellular organelles [29,30].

These findings suggested that the epoxy/FAS-TiO_2_/epoxy and epoxy/TiO_2_/epoxy coatings promote osteoblast growth. It is expected that the proposed approach to bio-functionalize and induce the antibacterial properties of a stainless-steel surface advance the development of new materials/surfaces for diverse biomedical applications.

### 3.3. Antibacterial Properties

Surfaces with different wettability were exposed to *E. coli* in a TSB medium for 24 h. The antibacterial properties of the coatings were evaluated by plate counting of the adhered *E. coli* with and without washing and presented in Table 2. We observed that the number of *E. coli* colonies decreased with the increased hydrophobicity and surface roughness of the coating. In general, moderate hydrophobic or hydrophilic surfaces are more able to bind bacteria in comparison with superhydrophobic and superhydrophilic surfaces [13]. However, we were able to considerably lower the *E. coli* adhesion also on the moderately hydrophobic coating (*θ*^W^ = 120°) due to the lowered surface energy by fluorination of the TiO_2_ nanoparticles. In addition, the superior antibacterial properties of epoxy/ FAS-TiO_2_/epoxy coating against *E. coli* compared with the epoxy/TiO_2_/epoxy and pure epoxy coatings are also governed by an increased surface roughness, which significantly reduced the contact area for bacterial binding and therefore weakens the adhesion. Moreover, the epoxy coatings with the addition of TiO_2_ and FAS-TiO_2_ nanoparticles, compared with the pure epoxy coating, resulted in reduced *E. coli* adhesion due to the known antibacterial effect of anatase TiO_2_ [31]. The antimicrobial properties of TiO_2_ nanoparticles are attributed to the reactive oxygen species, O^2−^, OH and HO_2_ radicals, which are produced by redox reactions between the adsorbed species (such as water and oxygen) and electrons and holes photo-generated by the UV irradiation of the TiO_2_, even under both natural solar irradiation and ordinary room light [15]. In addition, the photo-oxidation efficacy is correlated with the TiO_2_ surface area, and can be increased by reducing the particle size to the nanoscale [15].

## 4. Conclusions

We prepared two types of sandwich-structured coatings, epoxy/TiO_2_/epoxy and epoxy/FAS-TiO_2_/epoxy, that were applied on the surface of AISI 316L stainless steel. Both coatings are characterised by unique surface properties that differ in terms of wettability and surface roughness. The hydrophilic epoxy/TiO_2_/epoxy coating prepared with as-received TiO_2_ nanoparticles forms a smoother surface compared with the hydrophobic epoxy/FAS-TiO_2_/epoxy coating characterised with FAS-functionalised TiO_2_ nanoparticles agglomerates. The biocompatibility was evaluated in terms of the cell adhesion, viability and cell morphology of the MG63 cells. We have shown that the cell attachment was significantly higher on the epoxy/FAS-TiO_2_/epoxy and epoxy/TiO_2_/epoxy coatings compared with the reference pure epoxy coating. Additionally, the amount of observed dead cells on both coatings with TiO_2_ nanoparticles was considerably lower compared with the pure epoxy coating. Moreover, the epoxy coatings with the addition of TiO_2_ and FAS-TiO_2_ nanoparticles compared with the pure epoxy coating resulted in a reduced *E. coli* adhesion due to the known antibacterial effect of the anatase TiO_2_. Finally, we were able to successfully improve the biocompatibility and antibacterial properties of the epoxy coating with the implemented as-received and FAS-TiO_2_ nanoparticles.

## Figures and Tables

**Table 1:** Surface properties of the epoxy, epoxy/TiO_2_/epoxy and epoxy/FAS-TiO_2_/epoxy coatings on AISI 316L stainless steel: Static water contact angles (*θ*^W^), surface energy (*γ*) and the average surface roughness (*S_a_*).

Coating	*θ*^W^ (^o^)	*γ* (mN/m)	*S_a_* (nm)
epoxy	75 ± 1	38.6 ± 0.1	50 ± 5
epoxy/TiO_2_/epoxy	83 ± 1	33.7 ± 0.1	320 ± 20
epoxy/FAS-TiO_2_/epoxy	120 ± 3	11.6 ± 0.1	600 ± 30

**Table 2:** Number of colony-forming units of *E. coli* after 24-h incubation on epoxy, epoxy/FAS-TiO_2_/epoxy and epoxy/TiO_2_/epoxy coatings, before and after washing.

Coating	Colony Forming Units		*θ*^W^ (°)	*γ* (mN/m)
	No Wash	After wash		
Epoxy	countless	97	75 ± 1	38.6 ± 0.1
Epoxy/TiO_2_/epoxy	countless	47	83 ± 1	33.7 ± 0.1
Epoxy/FAS-TiO_2_/epoxy	70	7	120 ± 3	11.6 ± 0.1
